# The Proposed New York State Nurses’ Association

**Published:** 1901-04

**Authors:** 


### THE PROPOSED NEW YORK STATE NURSES' ASSOCIATION.

THE progressive spirit so active in the medical profession has incited the allied professions to almost equal activity regarding the fitness and qualification of its members, so that in New York State at the present time the Regents of the University decree who shall practice medicine, pharmacy, dentistry and veterinary science. That the regents, through the various state examining boards, have done their work honestly and courageously, no one will controvert, and that the allied professions stand on a higher plane than ever before no one will dare gainsay.

Allied to the medical profession, dependent and interdependent, stands the nursing profession, composed at the present time of a heterogeneous body of women, some well qualified, others poorly, and some possessing no qualifications except a diploma and uniform. Just as the medical profession recognised their once chaotic condition, so do the progressive nurses recognise theirs, and a movement is on foot to organise a New York State Nurses' Association. Such an organisation is intended to admit any nurse holding a diploma from a recognised reputable school, connected with a hospital, to active membership. Once organised and well established, and as Miss Sylveen Nye so aptly says, "having learned and proven that we know exactly what we want, then we may ask the legislature to pass a measure incorporating the following features: Uniform entrance examinations; uniform curriculum; uniform final examinations conducted by the regents, and that no hospital be allowed to maintain a training school unless it provides, either by its own or through combination with another, suitable clinical material."

The movement has started in the right way and needs the coöperation of every honest, intelligent nurse, who desires improvement in her chosen profession and security for herself. Before legislation can be demanded organisation and unity must be perfected, and then, with the approval and assistance of the medical profession, a nurses' licensing act can be passed and placed upon the statute book. Such a law would accomplish what has been so sadly needed: uniform qualification, uniform standard of training, the legislation of the title of "trained nurse," and what is most important, the best possible nursing for all classes.

The JOURNAL hopes to see such an organisation effected and congratulates the Buffalo nurses on taking the initiative in such a meritorious undertaking.

---

THE condition of the streets in Buffalo during the past few weeks has been far and away the worst in many years. Since smooth pavements have become the rule, the good care taken of them and the resulting cleanliness have been our pride and boast. Every visitor has complimented us in this respect and departed with a jealousy almost envious in character. The accumulation of snow and ice and the filth thus invited has recently placed us, more's the pity, in anything but an enviable attitude. Dr. Ernest Wende, the health commissioner, however, has solved the problem and the board of public works, rather than be indicted as a nuisance at the bar of public opinion, finally cleaned up the principal streets.

---

THE anti-spitting ordinance, after going through its various stages of struggle in committees and before both houses of the city legislature, where hearings were held, finally became a law. This is another triumph for the health department, which was seconded by physicians and women in large numbers,—all of whom are in favor not only of the prevention of disease, but of a wholesome cleanliness that should always prevail in every enlightened community.

---

THE ventilation of school buildings is one of the important questions that engages the attention of sanitarians and public health officers at the present time. In Buffalo it is a very live question, in view of the fact that the ventilation in school houses here is notoriously defective and sadly falls short of meeting the requirements of scientific modern sanitation. The school board, under the leadership of Dr. P. W. Van Peyma, chairman, is investigating the subject with a view to improvement, and the work of the board in this direction is ably seconded by Dr. Henry R. Hopkins, chairman of the committee on hygiene of the Medical Society of the State of New York. It is to be hoped that the efforts of these gentlemen will result in correcting an evil that is far reaching on the future health and lives of children of school age.

THE *New York Tribune* is always upon the side of right when the subject of professional standards is up for discussion. It does not favor lowering them in any respect, but if any change is desirable it advocates their advancement. It has recently presented strong arguments in its editorial columns against favorable action on certain bills before the present legislature, which the members would do well to heed. Still more recently, it has published an excellent cartoon, drawn by Mr. Leon Barritt, that is more forceful than any written argument against such harmful legislation. Through the courtesy of the *Tribune* we are enabled to republish this telling picture elsewhere in this magazine.

